# Are saliva and bile sources of oxalate secretion into the human gastrointestinal tract?

**DOI:** 10.14814/phy2.70827

**Published:** 2026-04-16

**Authors:** Emma Earhart, Thomas Tielleman, John Knight, Jonathan M. Whittamore

**Affiliations:** ^1^ Charles and Jane Pak Center for Mineral Metabolism and Clinical Research UT Southwestern Medical Center Dallas Texas USA; ^2^ Department of Internal Medicine, Division of Digestive and Liver Diseases UT Southwestern Medical Center Dallas Texas USA; ^3^ Department of Urology University of Alabama at Birmingham Birmingham Alabama USA

**Keywords:** salivary glands, Slc26a6

## Abstract

Elevated urine oxalate (hyperoxaluria) is a risk factor for kidney stones. Normally eliminated by the kidneys, oxalate originates from endogenous metabolism and dietary absorption but secretion into the intestine remains an open question. We considered saliva and bile as two potential sources. Previous studies report ≥100 μmol/L oxalate in saliva, far exceeding blood (1–3 μmol/L), implying secretion. With the liver being the main site of oxalate synthesis, bile is another path into the intestine. We set out to (1) Verify these salivary oxalate concentrations and rates of secretion. (2) Measure oxalate concentrations in bile. Saliva was obtained from healthy volunteers and bile from patients undergoing endoscopic retrograde cholangiopancreatography. Oxalate was determined by commercial assay and ion chromatography coupled with mass spectrometry (IC‐MS). With the assay, salivary oxalate averaged 21 μmol/L. However, using IC‐MS, values were ~1 μmol/L, comparable to plasma. This disparity resulted from positive interference with the enzyme‐based assay by one or more constituents in saliva. The rate of salivary secretion thus proved negligible. Bile oxalate averaged 14 μmol/L, suggesting hepatocyte secretion but biliary clearance is minor (<5%), relative to urine. We conclude saliva and bile are insignificant sources of secretion and unnecessary for determining oxalate clearance.

## 1 INTRODUCTION

Oxalate is the salt‐forming anion of oxalic acid and a non‐functional metabolic end‐product. Oxalate is eliminated in the urine and is of relevance to human health as one of the most common constituents of kidney stones in the form of insoluble calcium oxalate (Ermer et al., 2023; Knoll et al., 2011; Lieske et al., 2014). The kidneys are responsible for clearing this waste product from the body and keeping circulating concentrations of oxalate low, between 1 and 3 μmol/L (Costello & Landwehr, 1988; Fargue et al., 2023; Harris et al., 2004; Hatch, 1990). Urine oxalate excretion is typically <450 μmol/day, with up to 50% of this amount originating from the diet and the remainder coming from endogenous synthesis, principally the liver (Fargue et al., 2023; Holmes et al., 1995, 2001). The gastrointestinal (GI) tract therefore occupies a central role in oxalate homeostasis through the absorption and excretion of dietary oxalate, where it is naturally present in many plants and plant‐based foods (Holmes & Kennedy, 2000; Libert & Franceschi, 1987; Siener et al., 2006) and oxalate degradation by the resident gut microbiota (Abratt & Reid, 2010; Allison et al., 1986; Daniel et al., 2021).

Studies with rodent models have demonstrated an appreciable secretion of endogenous oxalate into the GI tract, which can potentially make a significant contribution to its elimination from the body (Whittamore & Hatch, 2022). For humans, however, extra‐renal excretion is considered trivial based on intravenous infusions of either ^14^C‐oxalate (reviewed by Whittamore & Hatch, 2022) or ^13^C‐oxalate (Fargue et al., 2023) where, on average, over 95% of labeled oxalate was recovered unchanged in the urine. The only evidence for secretion into the human GI tract remains indirect. For instance, variable concentrations of oxalate (4–79 μmol/L) were measured in fluid collected along the intestine of surgical patients being fed parenterally (Hatch & Freel, 1995). In another study, fluid retrieved from the small intestine of fasted patients during endoscopy contained up to 191 μmol/L soluble oxalate (Reddy et al., 2016). These findings imply some secretion may occur during the inter‐digestive period. Aside from the intestinal epithelium, another source of oxalate may be saliva. Several studies report high oxalate concentrations in human saliva, in some cases exceeding 100 μmol/L (Mydlik & Derzsiova, 2008; Wahl & Kallee, 1994), which are far greater than blood levels (1–3 μmol/L), thus requiring vigorous active secretion by the salivary glands. Indeed, oxalate concentrations approached 200 μmol/L in saliva collected from the isolated, perfused mouse submandibular gland (Mukaibo et al., 2018). Although not appreciably deposited in human tartar or teeth (Vincent et al., 2021; Wahl & Kallee, 1994), others have found oxalate in sialoliths and dental calculus (Davidovich et al., 2009; Kraaij et al., 2018; Yamamoto et al., 1983). With as much as 1 L of saliva produced daily (Iorgulescu, 2009; Richardson & Feldman, 1986; Watanabe & Dawes, 1988), this may therefore represent a significant, previously unrecognized source of oxalate entering the upper GI tract.

Since the liver is a major site of endogenous oxalate synthesis, bile is another way oxalate can enter the intestine. Numerous metabolic waste products and xenobiotics are disposed of in bile such as cholesterol and bilirubin, but little attention has been given to whether this includes oxalate. In rats, 3–10% of intravenous ^14^C‐oxalate was cleared in bile (Brubacher et al., 1956; Sugimoto et al., 1993). While this indicates that a small fraction of exogenous oxalate circulating through the liver enters bile, it reveals nothing about the fate of intracellular oxalate generated *de novo* and how much hepatocytes secrete into the canaliculi. Oxalate is not a known constituent of gallstones, except in rare and unusual cases (Liaqat et al., 2020; Wolf et al., 1982). However, the high concentrations of bile acids present in bile could effectively bind calcium, thus restricting the formation of insoluble calcium oxalate (Saso et al., 2001). To the best of our knowledge, the oxalate content of bile has not previously been reported, but it may serve as a way for the liver to route oxalate directly into the small intestine and avoid systemic exposure to this potentially toxic metabolite. If biliary clearance were found to be significant, this could have implications for understanding the fate of endogenous oxalate and estimating rates of synthesis which are currently based on urinary excretion minus dietary absorption.

To determine whether saliva and bile represent potential sources of oxalate secretion into the human GI tract we set out to (1) Verify the reported concentrations of oxalate in saliva and estimate its rate of secretion. (2) Collect and measure oxalate in bile. Herein, we report on the collection and pre‐analytical processing of saliva and bile from human subjects for the measurement of oxalate. We also compared the enzymatic and ion chromatographic methods for detecting oxalate in these two body fluids.

## 2 MATERIALS AND METHODS

### 2.1 Saliva collection

Two sequential timed collections of whole saliva (one resting and one following mechanical stimulation) were made by 12 healthy adult volunteers (*N* = 6 female, *N* = 6 male) recruited from UT Southwestern Medical Center ranging in age from 21 to 69 years (BMI: 16.7–31.0 kg/m^2^). All samples were collected in the morning between 8 a.m. and 12 p.m. Volunteers were on self‐selected diets and not required to be fasted but instructed to abstain from eating or drinking, brushing their teeth, and/or using mouthwash within 45 min of collections, although water was allowed up to 10 min before. First, a non‐stimulated sample was collected using the passive drool method (Navazesh, 1993), where saliva was allowed to pool under the tongue and then transferred into a pre‐weighed tube. Participants were then allowed water, and a 10‐min break was permitted prior to making the second collection. To stimulate saliva production participants were asked to chew on 5 cm^2^ of Parafilm® (Bemis, Neenah, WI, USA) collecting the saliva into another pre‐weighed tube. The tubes were weighed to determine the volume and rate of saliva production.

### 2.2 Bile collection

As a “hidden” secretion (Boyer, 2013), bile is not readily accessible and obtaining samples of either hepatic or gallbladder bile from normal, healthy individuals is hard to justify (Strasberg et al., 1990). We therefore collected hepatic bile from 10 adult patients (*N* = 6 female, *N* = 4 male) undergoing endoscopic retrograde cholangiopancreatography (ERCP) at Parkland Memorial Hospital in Dallas, TX. Subjects ranged in age from 19 to 81 years and fasted for at least 8–12 h prior to the procedure. In all subjects an ERCP was indicated for post‐operative bile leak following fenestrated cholecystectomy for acute cholecystitis. Bile leak cases were deliberately selected to avoid gallstones or other causes of bile duct obstruction. After gaining access to the bile duct and prior to injecting contrast, 2–7 mL of bile was aspirated into a pre‐weighed tube, placed on ice and immediately transported to the lab for processing.

### 2.3 Processing saliva

Samples were held on ice in the dark and processed immediately following collection. Deproteinization was performed by ultrafiltration (Amicon Ultra, 0.5 mL capacity, 10 kDa MWCO; Merck‐Millipore, Cork, Ireland). To remove any contaminants, including oxalate, from the filters prior to use, the ultrafiltration devices were rinsed with 0.1 N NaOH and then twice with ultrapure (18 MΩ·cm) water, centrifuging at 15,000×*g* for 5 min and 4°C each time. After vortex‐mixing each sample, two identical aliquots were taken using a positive displacement pipette. To determine and correct for any losses of oxalate during ultrafiltration, ^14^C‐oxalate (0.4 μCi/mL saliva; specific activity 2.42 mCi/mmol; NEN, Boston, MA, USA; Cat. No. NEC074) was added to one aliquot which was then thoroughly mixed and an initial 3 × 10 μL samples taken for scintillation counting (LS 6500; Beckman Coulter Inc., Brea, CA, USA). Both aliquots of saliva were placed in a pre‐rinsed ultrafiltration device and centrifuged at 15,000×*g* for 45 min and 4°C. The ultrafiltrate was collected directly into 3 N hydrochloric acid (20 μL/mL saliva) which was added to the pre‐weighed outer collection tube prior to centrifugation. After weighing the ultrafiltrate to determine the volume recovered, 3 × 10 μL samples were taken from the aliquot containing ^14^C‐oxalate to calculate recovery (Equation 1):

(1) $${\%\;}^{14}C\;\text{oxalate recovered}=A_{f}/\left(A_{i}\times \left(V_{s}/\left(V_{s}-V_{a}\right)\right)\right)\times 100$$

Where *A*_*i*_ and *A*_*f*_ are the initial and final counts of ^14^C‐oxalate (cpm) before and after ultrafiltration, respectively. V_s_ and *V*_*a*_ are the volumes (mL) of saliva ultrafiltrate recovered and 3 N HCl added to the collection tube, respectively. Acidifying samples after deproteinization (rather than before) served to (1) Limit the possibility of oxalate binding to saliva proteins, as occurs in blood plasma proteins (Costello & Landwehr, 1988), potentially contributing to losses during ultrafiltration. (2) Maintain oxalate in its soluble form. (3) Restrict oxalogenesis by stabilizing any ascorbic acid present in saliva (Bates et al., 1972; Feller et al., 1975; Makila & Kirveskari, 1969), thus limiting its conversion to oxalate. The acidified ultrafiltrates were stored at −20°C until analysis.

### 2.4 Processing bile

Samples were held on ice in the dark and processed immediately following collection. Any sediment was first pelleted by centrifugation at 3500×*g* for 20 min at 20°C followed by acidification to pH ≤ 1 to irreversibly precipitate the majority of bile salts (Barthlen et al., 1994), which would otherwise interfere with the detection of oxalate. Prior to acidification, two aliquots of each sample were processed in parallel. One was used for measuring oxalate and the other aliquot was spiked with ^14^C‐oxalate to trace any losses when acidified and the bile salts precipitated. To the latter, 0.4 μCi of ^14^C‐oxalate (specific activity 2.42 mCi/mmol) was added per mL of bile and thoroughly mixed before an initial 3 × 10 μL samples taken for counting. Ice‐cold 3 N HCl (100 μL/mL bile) was then added to both aliquots, which were thoroughly vortex‐mixed and placed on ice in the dark for 15–20 min. After centrifugation at 15,000×*g* for 5 min at 4°C, the supernatant was collected and another 3 × 10 μL samples of the ^14^C‐oxalate containing aliquot were taken to determine recovery (Equation 2):

(2) $${\%\;}^{14}C\;\text{oxalate recovered}=A_{f}/\left(A_{i}/\left(\left(V_{b}–V_{c}+V_{a}\right)/V_{b}\right)\right)\times 100$$

Where *A*_*i*_ are the initial counts of ^14^C‐oxalate (cpm) prior to acidification. *V*_*b*_ and *V*_*a*_ are the volumes (mL) of bile and acid, respectively, and *V*_*c*_ the initial volume (30 μL) taken for scintillation counting. *A*_*f*_ are the final counts of ^14^C‐oxalate (cpm) recovered in the acidified bile. All acidified supernatants were stored at −80°C until analysis.

### 2.5 Oxalate analysis in saliva and bile

Oxalate was determined with the commercial urine oxalate assay kit utilizing the enzyme oxalate oxidase (Trinity Biotech, Wicklow, Ireland; Cat. No. 591‐D), and ion chromatography coupled with mass spectrometry (IC‐MS) (ThermoFisher Scientific, Waltham, MA, USA). For the enzyme assay, all samples were prepared based on the manufacturer's instructions using the included sample diluent and adding the pH adjustment and charcoal‐filtration steps. Oxalate was then measured in standards, samples and quality controls by following a more sensitive version of the assay described by Ladwig et al. (2005) but omitting the addition of sodium nitrite. The resulting standard curve was linear between 5 and 160 μmol/L. Oxalate was also measured in the same samples by IC‐MS, as previously described (Fargue et al., 2023). Acidified ultrafiltrates were diluted four‐fold in ^13^C_2_‐oxalate internal standard (final ^13^C_2_‐oxalate concentration, 5 μmol/L; Cambridge Isotope Laboratories Inc., Andover, MA, USA; Cat. No. CLM‐4449‐1). The IC‐MS instrumentation consisted of an ISQ‐EC single quadrupole mass spectrometer coupled to a Dionex ICS‐5000 ion chromatograph with refrigerated AS‐AP auto‐sampler (10 μL injection loop) and gradient pump. Oxalate was separated using an IonPac 5 μm AS22, 2 × 250 mm anion exchange column fitted with an AG22 guard column (2 × 50 mm) with ammonium carbonate as the mobile phase (20–33 mM linear gradient over 30 min), at 30°C with a flow rate 0.3 mL/min. The combination of IC with MS enhances sensitivity and specificity, and the stable‐isotope internal standard improves accuracy. Stand‐alone IC with suppressed conductivity detection was also adopted for the analysis of saliva, but oxalate levels were below the threshold for accurate quantification, thus IC‐MS was subsequently used. Briefly, oxalate measurement by IC only utilized an IonPac 5 μm AS29, 2 × 250 mm, anion exchange column fitted with an AG29 guard column (2 × 50 mm), and carbonate/bicarbonate eluent (3.6 mmol/L NaCO_3_/1.6 mmol/L NaHCO_3_) operated isocratically at 30°C and a flow rate of 0.25 mL/min.

### 2.6 Statistical analysis

The following data are presented as mean (SD). For saliva, independent *t*‐tests were used to determine differences between saliva type (passive drool and stimulated). The percentage recoveries of volume and ^14^C‐oxalate were arcsine‐transformed prior to analysis. Statistical analyses were performed using SigmaPlot v15.0 (Inpixon Inc., Palo Alto, CA) and the results accepted as significant at *p* ≤ 0.050.

## 3 RESULTS

### 3.1 Saliva

Table 1 summarizes the production, processing, and oxalate concentrations of saliva from 12 healthy male and female volunteers. The rate of production was almost 5 times greater while chewing, but neither sex nor the type of saliva (passive drool or mechanically stimulated) significantly affected the volume or ^14^C‐oxalate recovered following deproteinization and had no bearing on the resulting oxalate concentration. Overall, the recovery of ^14^C‐oxalate in the ultrafiltrate was high, averaging 89.5 (6.4) % for all 24 samples collected but ranged from 76.8 to 97.6%. For each sample, the concentration of oxalate was adjusted to account for this variability in recovery between individuals. There were, however, vast differences between the two analytical methods, with the enzyme assay reporting average values around 20 μmol/L compared to just 1 μmol/L by IC‐MS.

**TABLE 1:** A comparison of non‐stimulated (resting) and mechanically stimulated saliva collected from 12 healthy male and female volunteers summarizing the rate of production, the volume and ^14^C‐oxalate recovered following deproteinization by ultrafiltration, and the oxalate concentrations measured in the resulting ultrafiltrate using the enzyme‐based commercial assay kit and ion chromatography coupled with mass spectrometry (IC‐MS).

Saliva type	Production (mL/min)	Ultrafiltration		Oxalate^a^	
		Volume recovered (%)	^14^C‐oxalate recovered (%)	Enzyme assay (μmol/L)	IC‐MS (μmol/L)
Non‐stimulated (resting)	0.21 (0.12)	85.8 (12.0)	87.0 (7.6)	21.43 (4.07)	1.10 (0.87)
Stimulated (mastication)	0.95 (0.71)*	92.4 (4.3)	92.0 (3.6)	20.22 (3.80)	0.95 (0.66)
*p*‐value	<0.001	0.052	0.087	0.459	0.705

*Note*: Data are mean (SD) for *n* = 12 of each saliva type.

^a^ Corrected based on the losses of ^14^C‐oxalate from each individual sample incurred during ultrafiltration.

* Significantly different from non‐stimulated (resting) saliva, *p* ≤  0.050.

**FIGURE 1:**
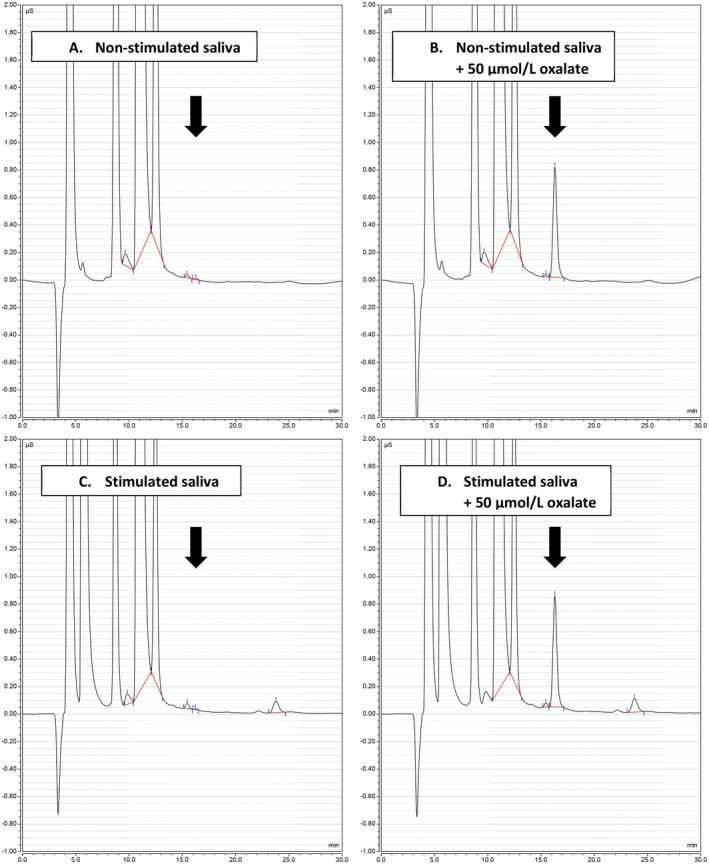
Representative chromatograms illustrating the oxalate content of human saliva was negligible using ion chromatography with conductivity detection (μS) in resting, non‐stimulated (Panel a) and stimulated (Panel c) samples of whole saliva collected from a single study participant. Each sample was divided into two and potassium oxalate was added to one aliquot to a final concentration of 50 μmol/L. Both aliquots were then processed in parallel, as described in the Materials and Methods. The corresponding chromatograms of the spiked aliquots are shown in Panels b (non‐stimulated) and d (stimulated). The oxalate peak with a retention time of 16.2–16.5 min is marked by an arrow.

Representative ion chromatograms of saliva ultrafiltrate are presented for a single subject to illustrate that the presence of the oxalate anion was negligible at the anticipated elution time of 16.2–16.5 min (Figure 1a,c). Repeating this analysis on a separate aliquot of the same sample spiked with 50 μmol/L potassium oxalate confirmed this (Figure 1b,d), indicating that the high concentrations determined by enzyme assay were false. To confidently identify and quantify the low levels of oxalate, we used IC‐MS, where concentrations ranged from 0.40 to 3.60 μmol/L across all 24 samples. Using these values and the corresponding rates of saliva production, oxalate secretion at rest was 0.013 (0.013) μmol/h (*n* = 12), increasing significantly to 0.084 (0.100) μmol/h (*n* = 12) when chewing (*p* < 0.001).

### 3.2 Bile

The characteristics of each patient undergoing ERCP, the volume of bile collected, ^14^C‐oxalate recovery following pre‐analytical acidification, and the concentrations of oxalate measured by IC‐MS are presented in Table 2. Upon review of their relevant medical history, none of the subjects had been diagnosed with any form of GI or liver disease known or suspected to influence oxalate handling. Patient #1 had undergone surgery to repair a gastric perforation due to a peptic ulcer, and another (patient #3) was in remission from hepatitis C. There was no prior history of symptomatic kidney stones and eGFR was normal (>90 mL/min/1.73 m^2^), with the exception of patient #10 (59 mL/min/1.73 m^2^). The majority (80%) were overweight based on BMI (>25 kg/m^2^), and the most common comorbidities were hypertension (*n* = 3), hyperlipidemia (*n* = 2), diabetes (*n* = 3), and obesity (*n* = 1). No subjects were on antibiotics or reported taking any probiotic, vitamin C or calcium supplements at the time of the procedure. Prescribed medications taken by these patients included albuterol (*n* = 1), amlodipine (*n* = 2), atorvastatin (*n* = 2), allopurinol (*n* = 1), empagliflozin (*n* = 1), hydrochlorothiazide (*n* = 2), insulin (*n* = 1), losartan (*n* = 1), metformin (*n* = 1), and norethindrone (*n* = 1). The volume of bile collected ranged from 2.1 to 6.8 mL, averaging 3.5 (1.3) mL. There were no appreciable losses of ^14^C‐oxalate when bile salts were precipitated with a mean recovery of 100.9 (3.5) %, (range 92.1–104.1%). The resulting oxalate concentrations were therefore not adjusted. The samples were initially analyzed using the commercial oxalate assay as described in the Materials and Methods (Section 2.5). While this assay was linear between 5 and 160 μmol/L, each sample ended up being diluted 2.5‐fold in preparation, and only the bile from patient #8 had measurable oxalate (8.5 μmol/L) by this method. The remainder possessed oxalate levels <5 μmol/L. Sufficient volume was leftover for 8 out of 10 samples for analysis by IC‐MS, which is capable of reliably detecting 0.5 μmol/L oxalate. The concentration in these remaining samples presented in Table 2 averaged 13.6 (1.9) μmol/L.

**TABLE 2:** The volume of bile collected, recovery of ^14^C‐oxalate after processing, and subsequent oxalate concentrations determined by ion chromatography coupled with mass spectrometry (IC‐MS) for 10 patient cases of post‐cholecystectomy bile leak.

Patient	Age (years)	Sex	BMI (kg/m^2^)	eGFR (mL/min/1.73 m^2^)	Volume collected (mL)	^14^C‐oxalate recovered (%)	Oxalate (μmol/L)
1	62	M	23.8	103	6.76	102.3	13.7
2	36	F	35.2	120	4.50	98.4	11.5
3	38	F	32.6	117	3.00	103.9	14.9
4	44	M	35.2	108	3.15	101.5	‐
5	46	F	29.9	119	2.86	100.4	12.5
6	58	M	24.8	105	3.52	104.1	12.0
7	19	F	29.1	121	2.68	102.3	‐
8	49	M	30.0	103	3.23	100.6	14.3
9	49	F	31.6	119	2.06	103.1	12.7
10	81	F	26.0	59	3.48	92.1	17.4
					3.5 (1.3)	100.9 (3.5)	13.6 (1.9)

*Note*: Bile was collected from the bile duct during endoscopic retrograde cholangiopancreatography (ERCP), prior to the injection of contrast. The age, sex, BMI, and eGFR of each patient are also presented. See Section 3.2 for further details.

## 4 DISCUSSION

The human GI tract does not have a recognized role in the secretion and elimination of endogenous oxalate. However, concentrations up to 100 times higher than blood have been reported in saliva of humans (Mydlik & Derzsiova, 2008; Wahl & Kallee, 1994), and mice (Mukaibo et al., 2018), where the salivary glands of the latter were shown to actively secrete oxalate. The liver is a major site of endogenous oxalate synthesis, but the amount cleared in the bile and entering the small intestine is unknown. The results of this study suggest saliva and bile are only minor sources of oxalate and do not make a meaningful contribution to its clearance from the body.

### 4.1 Oxalate in saliva

In two of three previous studies, oxalate in human saliva was ≥100 μmol/L when measured using the commercial urine oxalate assay kit (Table 3). Adopting the same enzyme‐based assay, we found concentrations were closer to 20 μmol/L (range 15.3–26.7 μmol/L), yet these still far exceed levels in plasma (1–3 μmol/L). Attempting to verify this result using IC with conductivity detection, we discovered no such concentrations were present (Figure 1). This profound disparity between methods contrasts with the analysis of saliva from the perfused mouse submandibular gland, where 210 μmol/L was reported by the enzymatic urine oxalate assay and subsequently confirmed by IC (Mukaibo et al., 2018). To correctly identify and quantify these low levels of oxalate in human saliva, we adopted IC‐MS and determined concentrations were very similar to blood, averaging just 1 μmol/L. These plasma‐like concentrations are consistent with an unpublished report of 2.7 μmol/L in human saliva analyzed by HPLC (Omori et al., 1986). Using the enzymatic assay, comparable levels of 3.4 μmol/L were measured in healthy control children by Davidovich et al. (Davidovich et al., 2009) (Table 3), noting oxalate was undetectable in 2 of the 5 participants. Interestingly, compared to these normal controls, this latter study did show 3‐fold higher concentrations in saliva collected from a small number of pediatric patients on dialysis. If the oxalate concentrations circulating in blood are accurately reflected in saliva, it could serve as a rapid, non‐invasive approach to regularly monitor plasma oxalate in cases of renal dysfunction such as chronic kidney disease (CKD) and Primary Hyperoxaluria.

**TABLE 3:** A comparison of oxalate concentrations in whole human saliva determined using the commercial urine oxalate assay kit based on the enzyme oxalate oxidase.

References	Subjects (*n*)	Oxalate (μmol/L)	Type of saliva	Pretreatment
Wahl & Kallee (1994)	Males (41)	100 (90)	Non‐stimulated	Lyophilized, acidified, heated
	Females (40)	180 (170)		
Mydlik & Derzsiova (2008)	Control (13)	128 (19)	Stimulated	Not reported
	CKD (24)	135 (24)		
Davidovich et al. (2009)	Control (pediatric) (3)	3.4 (1.3)	Non‐stimulated	None
	Dialysis (pediatric) (4)	11.1 (2.8)		
Present work^a^	Males and females (12)	21.4 (4.1)	Non‐stimulated	Ultrafiltered, acidified
		20.2 (3.8)	Stimulated	

*Note*: These values are presented alongside those determined by the current study using both the enzymatic assay and ion chromatography coupled with mass spectrometry (IC‐MS). Values are mean (SD).

^a^ Oxalate concentrations were corrected for losses incurred during ultrafiltration.

Saliva is a dilute fluid made up of 99% water by weight but with a complex composition. Production is a two‐step process beginning with secretion of an isotonic fluid by the acinar cells of the salivary glands which is plasma‐like in terms of sodium (Na^+^) and chloride (Cl^−^). This primary secretion moves out of the acinus and along the salivary ducts where its ionic composition is modified by the duct cells as they re‐absorb Na^+^ and Cl^−^ while secreting potassium and bicarbonate (HCO_3_^−^), resulting in a dilute, hypotonic saliva entering the oral cavity (Chibly et al., 2022; Ohana, 2015; Turner & Sugiya, 2002). The secretion of oxalate could potentially occur at any stage of this process. The Cl^−^/HCO_3_^−^ exchanger known as PAT‐1 (Putative Anion Transporter 1), encoded by gene Slc26a6, is an oxalate transporter which, in addition to prominent expression in the small intestine and pancreas, has also been localized to the apical membrane of duct cells in the mouse parotid gland (Ko et al., 2004). In the mouse submandibular gland, PAT‐1 has a major role in oxalate secretion across the apical membrane of acinar cells, where the resulting saliva contained ~200 μmol/L (Mukaibo et al., 2018). This likely reflects a unique, species‐specific difference in the oxalate transporting capabilities of rodent salivary glands compared to those of humans where the distribution of PAT‐1 has not been described. Having determined that both basal and stimulated saliva contains 1 μmol/L, the accompanying rates of oxalate production calculated from Table 1 can be used to estimate total daily secretion. Excluding the very low volumes of saliva produced during sleep (Lear et al., 1965; Schneyer et al., 1956), over the course of 16 waking hours, with ~1 h spent eating a total of 0.3 μmoles of oxalate (0.013 μmol/h × 15‐h + 0.084 μmol/h) would be produced, corresponding to <0.2% of typical urinary excretion (150–450 μmoles/day). We therefore conclude saliva is not a source of oxalate secretion into the human GI tract.

The disparity between the commercial urine oxalate assay and IC‐MS may be the result of one or more compounds in saliva positively interfering with the coupled enzyme reactions of the former. In this assay, oxalate oxidase first converts oxalate to CO_2_ and hydrogen peroxide (H_2_O_2_) which subsequently undergoes oxidative coupling with the chromogens 3‐methyl‐2‐benzothiazolinone hydrazone (MBTH) and 3‐(dimethylamino) benzoic acid (DAB), catalyzed by horseradish peroxidase (HRP), to produce an indamine dye. The intensity of the color change is therefore directly proportional to the amount of H_2_O_2_ (and oxalate) present (Laker et al., 1980).

$$C_{2}O_{4}H_{2}+O_{2}\;\overset{\text{Oxalate oxidase}}{\to }\;{\text{2CO}}_{2}+H_{2}O_{2}$$

$$H_{2}O_{2}+\text{MBTH}+\mathrm{DAB}\;\overset{\text{Horseradish peroxidase}}{\to }\;\text{Indamine}\;\mathrm{dye}+H_{2}O$$

Of the potential interferants with these reactions, the antioxidant ascorbate was the most potent (Laker et al., 1980) competing with HRP for H_2_O_2_ causing oxalate to be underestimated (Kasidas & Rose, 1985). Ascorbic acid is also unstable in alkaline solutions and will degrade (non‐enzymatically) to oxalate (Chalmers et al., 1985; Mazzachi et al., 1984; Robertson & Scurr, 1984). Precautions were therefore taken to avoid oxalogenesis by acidifying samples and filtering through activated charcoal (Section 2.5). The ion chromatograms in Figure 1a,c show oxalate was virtually undetectable by IC alone, thus ascorbate is an unlikely source of oxalate. An alternative interferant might be H_2_O_2_, which is a normal constituent of human saliva (Nishioka et al., 1996; Pruitt et al., 1983). In sufficient amounts, H_2_O_2_ would provide additional substrate for HRP causing oxalate to be overestimated. In the oral cavity, salivary peroxidases use H_2_O_2_ to oxidize thiocyanate to hypothiocyanite, a potent antimicrobial agent (Ihalin et al., 2006; Tenovuo & Pruitt, 1984). This innate defense mechanism also prevents the toxic build‐up of H_2_O_2_, thus making reliable measurements challenging (Redanz et al., 2018). Using the equilibrium expression for thiocyanate peroxidation, H_2_O_2_ concentrations were estimated to be 8–13 μmol/L (Pruitt et al., 1986). This may therefore explain some of the erroneously high oxalate values presented in Table 1, which ranged between 15 and 27 μmol/L. However, this remains speculation and the identity of the constituent(s) responsible is unclear since interference from H_2_O_2_, or indeed any other oxidizing agents, should have been minimized or eliminated by the charcoal filtration step of the assay.

### 4.2 Oxalate in bile

**FIGURE 2:**
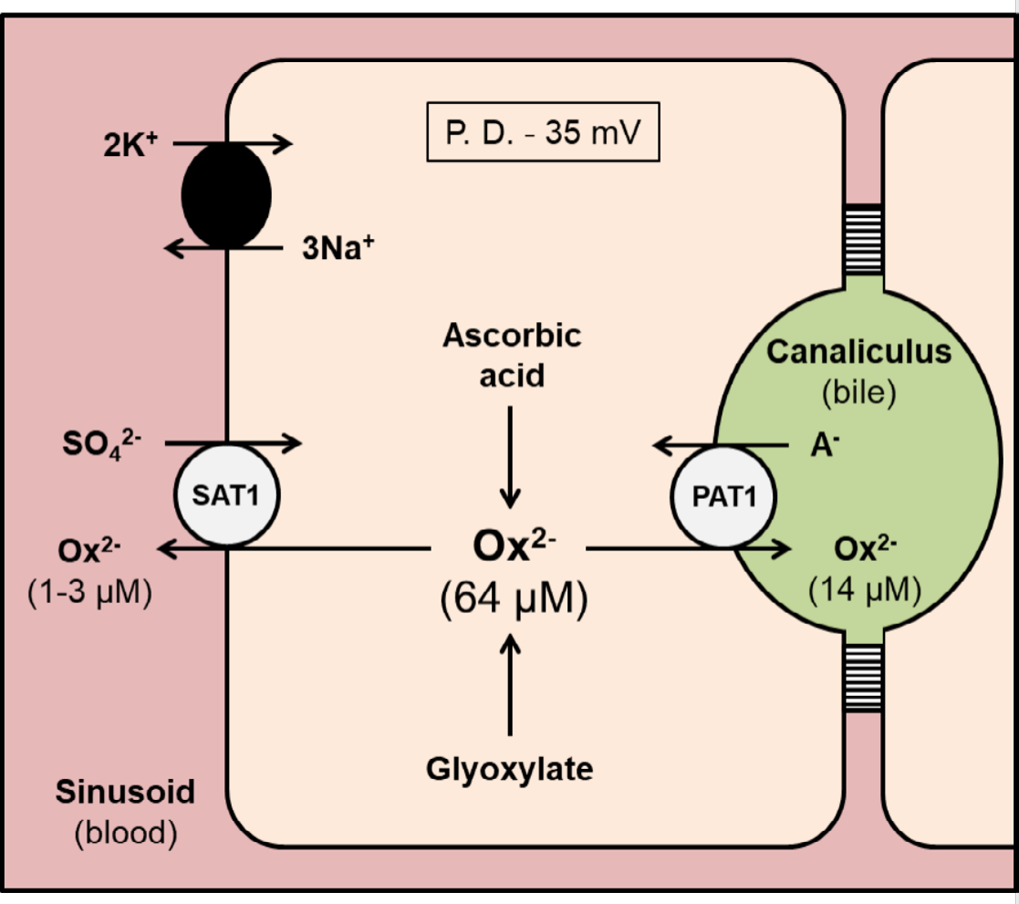
Proposed model of oxalate secretion by hepatocytes. Oxalate (Ox^2−^) is generated from the detoxification of glyoxylate and non‐enzymatic breakdown of ascorbic acid. The multifunctional anion exchanger PAT‐1 (Slc26a6) is expressed at the canalicular membrane of (rat) hepatocytes and may be involved in secreting cytosolic oxalate into the bile (see Section 4.2 for further details). The sulfate anion transporter (SAT‐1; Slc26a1) has been localized to the opposing sinusoidal membrane of rodent hepatocytes where it is proposed to operate as a SO_4_^2−^/Ox^2−^ exchanger exporting intracellular oxalate into the blood (Breljak et al., 2015; Brzica et al., 2009; Dawson et al., 2010; Quondamatteo et al., 2006). The oxalate concentration within human hepatocytes was estimated by Baker et al. (2004), and in hepatic bile by the present study (Table 2).

Oxalate generated by hepatocytes is exported into the blood where it is filtered and secreted by the kidneys and eliminated in the urine (Ermer et al., 2023; Fargue et al., 2023). Yet, there has been no consideration of how much might be directed into the small intestine *via* bile. The average concentration of oxalate in hepatic bile obtained from patients undergoing ERCP was 13.6 μmol/L (Table 2). With humans producing 500–600 mL of bile each day (Boyer, 1987), this equates to ~8 μmol/24‐h, representing 2–5% of total urinary excretion (150–450 μmol/24‐h). This is comparable to the 2.7% of intravenous ^14^C‐oxalate eliminated in the bile of rats relative to urine (Sugimoto et al., 1993), suggesting biliary clearance is also not a significant pathway for oxalate disposal in humans. Nevertheless, there must be some oxalate secretion across the canalicular membrane to accumulate concentrations in bile up to 10‐times higher than blood. With a transepithelial potential difference of −5 mV (with respect to the blood) across the tight junctions between hepatocytes (Graf et al., 1987), simple paracellular flux into the canalicular lumen is not thermodynamically feasible, yet there is tremendous scope for transcellular secretion (Figure 2). With an estimated cytosolic concentration of 60 μmol/L oxalate in human hepatocytes (Baker et al., 2004), and a potential difference across the canalicular membrane of 35 mV (cell negative) (Moule & McGivan, 1990), the electrochemical driving force acting on intracellular oxalate is strongly directed outward into bile. A possible candidate transporter facilitating this export is PAT‐1, the multifunctional anion exchanger with a major role in oxalate secretion by (mouse) salivary glands (Mukaibo et al., 2018), small intestine (Freel et al., 2006; Jiang et al., 2006), and renal proximal tubule (Knauf et al., 2019). PAT‐1 has also been localized to the canalicular membrane of rat hepatocytes (Karaica et al., 2018), but its role there has not been examined, and our findings suggest it would only make a minor contribution to oxalate clearance, at least when kidney function is normal. However, in CKD and end‐stage renal failure, biliary secretion may gain greater prominence. For example, the fraction of oxalate cleared in bile from rats after bilateral nephrectomy increased more than 6‐fold from 2.7% to 17.6%, supporting the existence of an adaptive shunt from urine to bile (Sugimoto et al., 1993). This is consistent with previous rodent models of CKD demonstrating a shift in oxalate excretion from the kidney to the gut (Costello et al., 1992), resulting in (secondary) active transcellular oxalate secretion by the distal colon (Hatch et al., 1994), where PAT‐1 is absolutely required for increased elimination in the feces (Neumeier et al., 2020). Whether a similar adaptation occurs in human CKD is unknown. However, it is notable that patient #10, with mild‐to‐moderate loss of kidney function (eGFR = 59 mL/min/1.73 m^2^), equivalent to stage 3a CKD, had a bile oxalate concentration approximately one‐third higher (17.4 μmol/L) compared to patients with normal eGFR (Table 2).

## 5 CONCLUSION

This study set out to determine whether saliva and bile are potential sources of oxalate in the human GI tract. We were unable to verify the high oxalate concentrations (≥100 μmol/L) in saliva reported by other investigators and suggest the commercial enzymatic assay kit used in these prior studies falsely overestimated oxalate content. Saliva collected from healthy adult volunteers contained ~1 μmol/L oxalate (range 0.4–3.6 μmol/L) as determined by IC‐MS, with no differences in concentration between non‐stimulated and stimulated saliva. In relation to the daily rate of saliva production, we estimate the total output of oxalate is negligible, just 0.3 μmol/24‐h, representing <0.2% of daily urinary excretion. Since the liver is the main contributor to endogenous oxalate synthesis, we also explored whether oxalate was eliminated in bile. We collected and analyzed hepatic bile from patients undergoing ERCP for bile leak following cholecystectomy and, using IC‐MS, measured concentrations ranging from 11.5 to 17.4 μmol/L. While the appearance of oxalate in bile likely involves (secondary) active secretion by hepatocytes, biliary clearance is projected to constitute a very minor fraction (2–5%) of oxalate excretion. However, bile may assume greater significance as an excretory route for oxalate with declining kidney function and in renal failure.

## AUTHOR CONTRIBUTIONS

**Emma Earhart:** Data curation; investigation; methodology. **Thomas Tielleman:** Conceptualization; investigation; methodology; resources. **John Knight:** Conceptualization; data curation; formal analysis; funding acquisition; investigation; methodology; resources; validation. **Jonathan M. Whittamore:** Conceptualization; data curation; formal analysis; funding acquisition; investigation; methodology; project administration; supervision; validation; visualization.

## FUNDING INFORMATION

This work was supported by the Charles and Jane Pak Center for Mineral Metabolism and Clinical Research at UT Southwestern, and the National Institutes of Health/NIDDK grant R01DK125824 (Knight).

## CONFLICT OF INTEREST STATEMENT

The authors declare no conflicts of interest.

## ETHICS STATEMENT

This study was performed in accordance with the ethical standards of UT Southwestern Medical Center and the principles of the 1964 Helsinki Declaration and its later amendments. Approval for these study protocols was obtained from UT Southwestern Medical Center Institutional Review Board (Protocol numbers: STU‐2022‐1099 and STU‐2023‐0723).

## CONSENT TO PARTICIPATE

Informed verbal consent was obtained from all individual participants included in the study.

## Data Availability

The data obtained and analyzed in this study are available upon request to the corresponding author.
